# A Restriction Boundary-Based Coronal Plane Alignment of the Knee (CPAK) Classification for Restricted Kinematic Alignment Total Knee Arthroplasty

**DOI:** 10.7759/cureus.72244

**Published:** 2024-10-23

**Authors:** Shotaro Araki, Takafumi Hiranaka, Takaaki Fujishiro, Koji Okamoto

**Affiliations:** 1 Orthopedic Surgery and Joint Surgery Center, Takatsuki General Hospital, Takatsuki, JPN

**Keywords:** lateral distal femoral angle (ldfa), medial proximal tibial angle (mpta), restricted kinematic alignment (rka), restriction boundary-based cpak, the coronal plane alignment of the knee (cpak)

## Abstract

Background

Coronal plane alignment of the knee (CPAK) classification was proposed as a means of understanding the knee phenotype in leg alignment and joint line obliquity (JLO). However, when it is adapted to restricted kinematic alignment total knee arthroplasty (rKA-TKA), the boundaries of CPAK and those of rKA-TKA phenotype are different. We therefore reappraise the boundary between the CPAK classification and restriction protocol and propose a restriction boundary-based CPAK (Rb-CPAK).

Methods

Between May 2020 and March 2022, 143 knees in 95 patients underwent rKA at our institution and were included in this study. In Rb-CPAK, we set the following ranges: 6° varus to 3° valgus for arithmetic hip-knee-ankle angle (aHKA), 0° to 6° varus for the medial proximal tibial angle (MPTA), 0° to 5° valgus for the lateral distal femoral angle (LDFA), and 169° to 180° for JLO. The pre- and postoperative alignments were classified using the original CPAK and Rb-CPAK.

Results

There were significant differences in pre- and postoperative distributions between original CPAK and Rb-CPAK (p < 0.0001). Postoperative Rb-CPAK primarily led to neutral aHKA (116 of 143 knees), and decreased MPTA varus (pre: 83.9 ± 3.4, post: 87.0 ± 2.3, p < 0.0001) and stable LDFA values (pre: 88.7 ± 3.1, post: 88.5 ± 2.7, p = 0.4) were observed. Among cases with neutral JLO, 78 knees required MPTA or LDFA corrections. Postoperatively, 67 (64%) out of 119 knees categorized as neutral JLO fell within MPTA and LDFA ranges.

Conclusion

The Rb-CPAK modification more effectively outlined knees that required restriction, and the restriction was properly performed compared with the original CPAK. However, JLO does not effectively indicate if a knee requires restriction or not, and thus individual evaluation of LDFA and MPTA might be necessary.

## Introduction

Total knee arthroplasty (TKA) is an effective treatment for end-stage knee osteoarthritis. Good long-term survival accounted for approximately 95% of 10-year survivorship and good clinical outcomes [1-3]. Good results have been obtained under the mechanical alignment (MA) concept, which comprises neutral leg alignment (0° of hip-knee-ankle angle [HKA]), mechanical component placement (perpendicular to the mechanical aces), and balanced gaps (parallel and same gaps in flexion and extension).

Kinematic alignment (KA) TKA was introduced by Howell and Hull [4]. The component is set along the joint surface respecting the three kinematic axes (cylindrical axis, patellar axis, and tibial rotation axis). As the components nearly replicate the patients' native articular surface, soft-tissue balancing can be natural, and soft-tissue release is rarely necessary [5,6]. On the other hand, component alignment is not always mechanical, and it sometimes reaches an extreme alignment that has been recognized as an outlier and can cause component loosening [7,8]. Although recent reports have shown that there is no increase in failure rate regardless of the component alignment [9, 10], it might still be a concern whether such placement is also safe in Asian patients [11], in whom constitutional varus is prevalent. Restricted KA (rKA) TKA has been introduced to avoid extreme alignment placement. In rKA-TKA, the KA approach is used within an alignment boundary; otherwise, components are placed in a defined alignment [12].

In rKA-TKA, it is important to understand the phenotype of the knee because the knee that requires restriction is easily recognized. The coronal plane alignment of the knee (CPAK) classification was proposed by MacDessi et al. to understand the knee phenotype in leg alignment and joint line obliquity (JLO) [13]. Using the lateral distal femoral angle (LDFA) and the medial proximal tibial angle (MPTA), arithmetic HKA (aHKA), representing global leg alignment, is calculated as MPTA - LDFA, and JLO is calculated as MPTA + LDFA. The aHKA is classified into varus (less than -2°), neutral (<±2°), and valgus (>2°), and JLO is categorized into apex-distal (<177°), neutral (177° to 183°), and apex-proximal (>183°). Based on the aHKA and JLO, a knee is classified from I to XI.

Although the CPAK is convenient for simply describing the phenotype of coronal alignment of patients’ knees, it is not useful if a knee requires restriction and undergoes the rKA-TKA properly. It is thought to be reasonable that the restriction should be decided based on a restriction boundary. This study therefore aims to modify the CPAK based on a restriction boundary (restriction boundary-based CPAK [Rb-CPAK]) classification determined by our restriction protocol. We evaluate the pre- and postoperative Rb-CPAK in patients who underwent rKA-TKA. We hypothesized that the Rb-CPAK more likely outlines the need for restriction and the results of restriction.

## Materials and methods

Patients

This retrospective study was performed according to the Declaration of Helsinki and approved by the Ethics Committee of our institution (2018-79). We recruited 164 knees in 105 consecutive patients who underwent rKA in our hospital between May 2020 and March 2022. We excluded four knees due to previous surgeries (two were high tibial osteotomy and two were total hip arthroplasty), one due to previous tibial plateau fracture, 14 knees due to inadequate radiography (mostly malrotation of the leg), and two due to the lack of radiography because of transfer to another department. Consequently, 143 knees in 95 patients (male: 33 knees in 21 patients; female: 110 knees in 74 patients) were finally included in this study. The average age was 74.0 ± 8.3 years, ranging from 55 to 93 years. Posterior cruciate ligament retention type TKA was performed using Persona (Zimmer Biomet, Warsaw, IN), GMK Sphere (Medacta, International AG, Castel San Pietro, Switzerland), and Vanguard ID (Zimmer Biomet, Warsaw, IN).

Surgical technique

**Figure 1:**
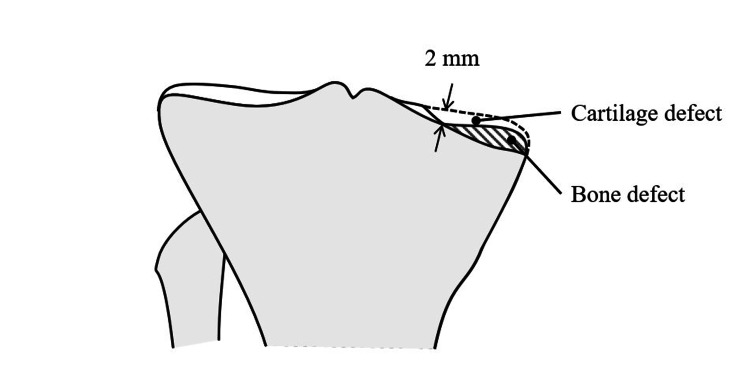
Tibial osteotomy The cutting level was adjusted at 10 mm below the lateral tibial articular surface using the stylus. The coronal slope was adjusted using another stylus set as 8 mm and pointed to the medial margin of the remnant cartilage at the base of the medial tibial spine. This figure was illustrated by us.

All surgeries were performed by the senior author (T.H.) or under his supervision. A modified subvastus approach was used in every case [14], and medial capsulotomy was used. After the osteophyte removal, distal femoral cuts, followed by posterior femoral cuts, were completed in the same thickness as the component, compensating for cartilage wear. For example, in medial osteoarthritis, the resection thickness of the distal medial and lateral femoral condyle was 7 mm and 9 mm, respectively, compensating for the cartilage defect by 2 mm. In most cases, the cartilage of the posterior condyle was not worn; the cutting thickness was 9 mm for each. Every cut bone thickness was measured using a caliper, and if the difference was more than 1 mm, a recut was made. As for the tibial side, the tibial slope was adjusted, referencing a pin penetrating the cutting slot of the tibial cutting guide or an angel wing by adjusting the inclination of the extramedullary rod. Then, using the stylus, the cutting level was adjusted at 10 mm below the lateral tibial articular surface. The coronal slope was adjusted using another stylus set at 8 mm and pointed to the medial margin of the remnant cartilage at the base of the medial tibial spine (Figure 1). This point is considered to be 2 mm below the original articular surface, even in cases with tibial bone defects. The medial inclination (varus angulation) can be excessive in some cases. In such cases, the alignment was adjusted by controlling the distal end of the intramedullary tibial rod so as to not exceed the lateral malleolus. Using this technique, the medial inclination was restricted approximately up to 5.5° based on a recent report [15]. The extension and flexion gaps were then evaluated using a spacer block, and a recut was made if there was an unacceptable imbalance.

Radiographic evaluation

**Figure 2:**
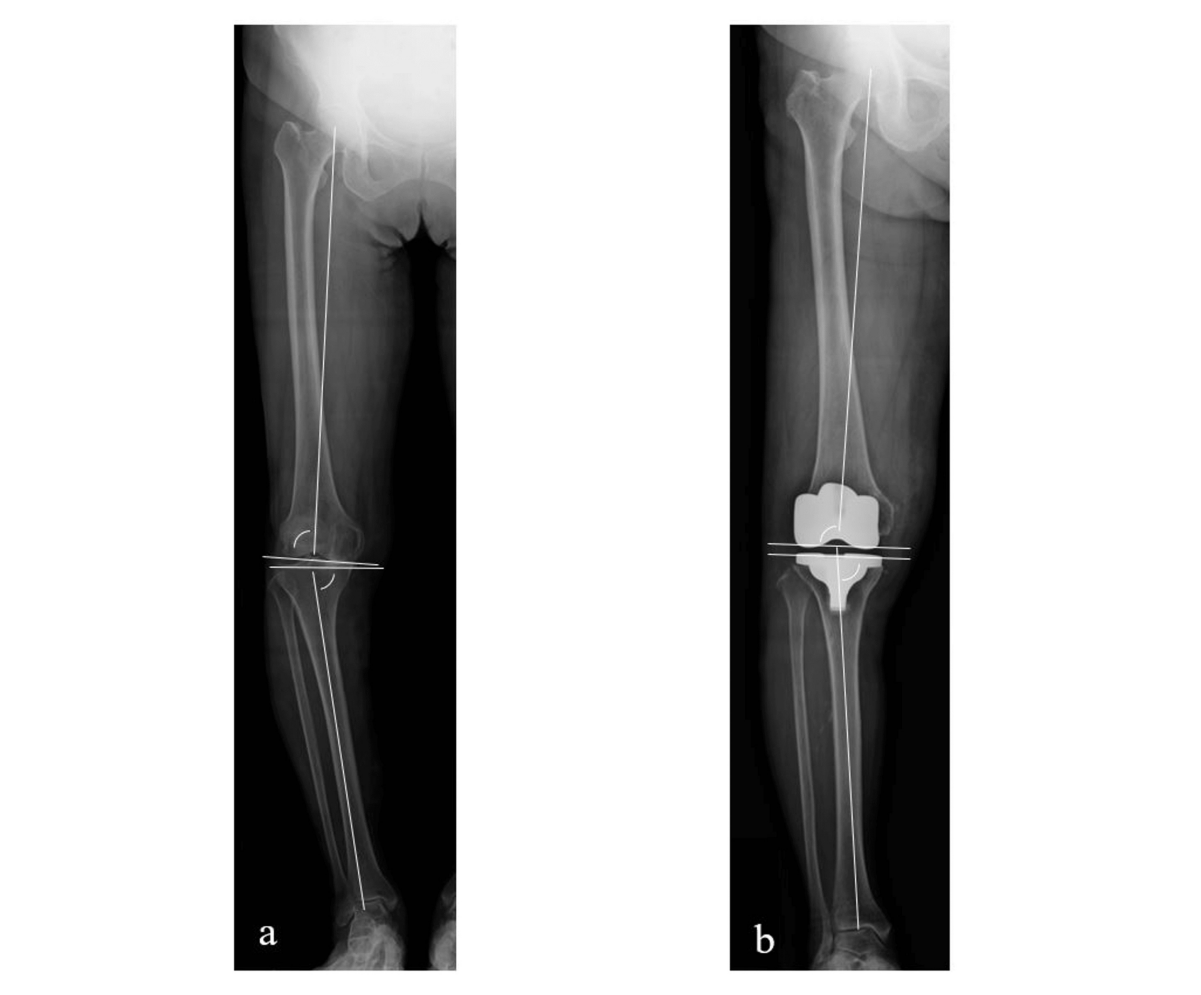
Pre- and postoperative MPTA and LDFA measurements (a) Preoperative LDFA was defined as the lateral angle between the mechanical femoral axis and the articular surface, and preoperative MPTA was defined as the medial angle between the mechanical tibial axis and the articular surface. (b) The component surfaces were considered to be the articular surface and to be parallel with the bony surface. LDFA and MPTA were therefore measured using the surface of the component and the axis of the femur and tibia. This figure was created by us. LDFA, lateral distal femoral angle; MPTA, medial proximal tibial angle

As a part of routine examinations, long-leg standing radiography was taken preoperatively and then two weeks postoperatively. The LDFA and MPTA were measured on the radiographs, and aHKA and JLO were calculated. The LDFA and the MPTA were measured as shown in Figure 2. Preoperative LDFA was defined as the lateral angle between the mechanical femoral axis and the articular surface, and preoperative MPTA was defined as the medial angle between the mechanical tibial axis and the articular surface (Figure 2a). After TKA, the component surfaces were considered to be the articular surface and to be parallel with the bony surface. LDFA and MPTA were therefore measured using the surface of the component and the axis of the femur and tibia (Figure 2b). Using these values, the original CPAK classification using the boundary by MacDessi et al. and the Rb-CPAK classification using our restriction boundary have been used.

Original coronal plane alignment of the knee (MacDessi et al.)

**Figure 3:**
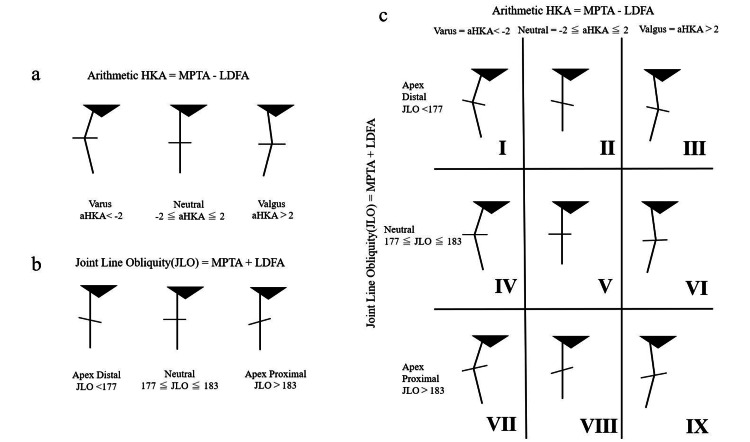
Coronal plane alignment of the knee (CPAK) (MacDessi et al.) (a) Category of aHKA. (b) Category of JLO. (c) coronal plane alignment of the knee classification. This figure was adapted from Vendittoli et al. [12]. HKA, hip-knee-ankle angle; JLO, joint line obliquity; LDFA, lateral distal femoral angle; MPTA, medial proximal tibial angle

In the CPAK, the leg alignment is expressed as the aHKA (MPTA - LDFA) and classified as varus, neutral or valgus (Figure 3a). The inclination of the joint line is articulated as the JLO (MPTA + LDFA) and categorized as apex distal, neutral, or apex proximal (Figure 3b). Combining the aHKA and JLO categories, all knees were classified into one of nine categories, from I to IX (Figure 3c).

Restriction boundary-based coronal plane alignment of the knee (Rb-CPAK)

**Figure 4:**
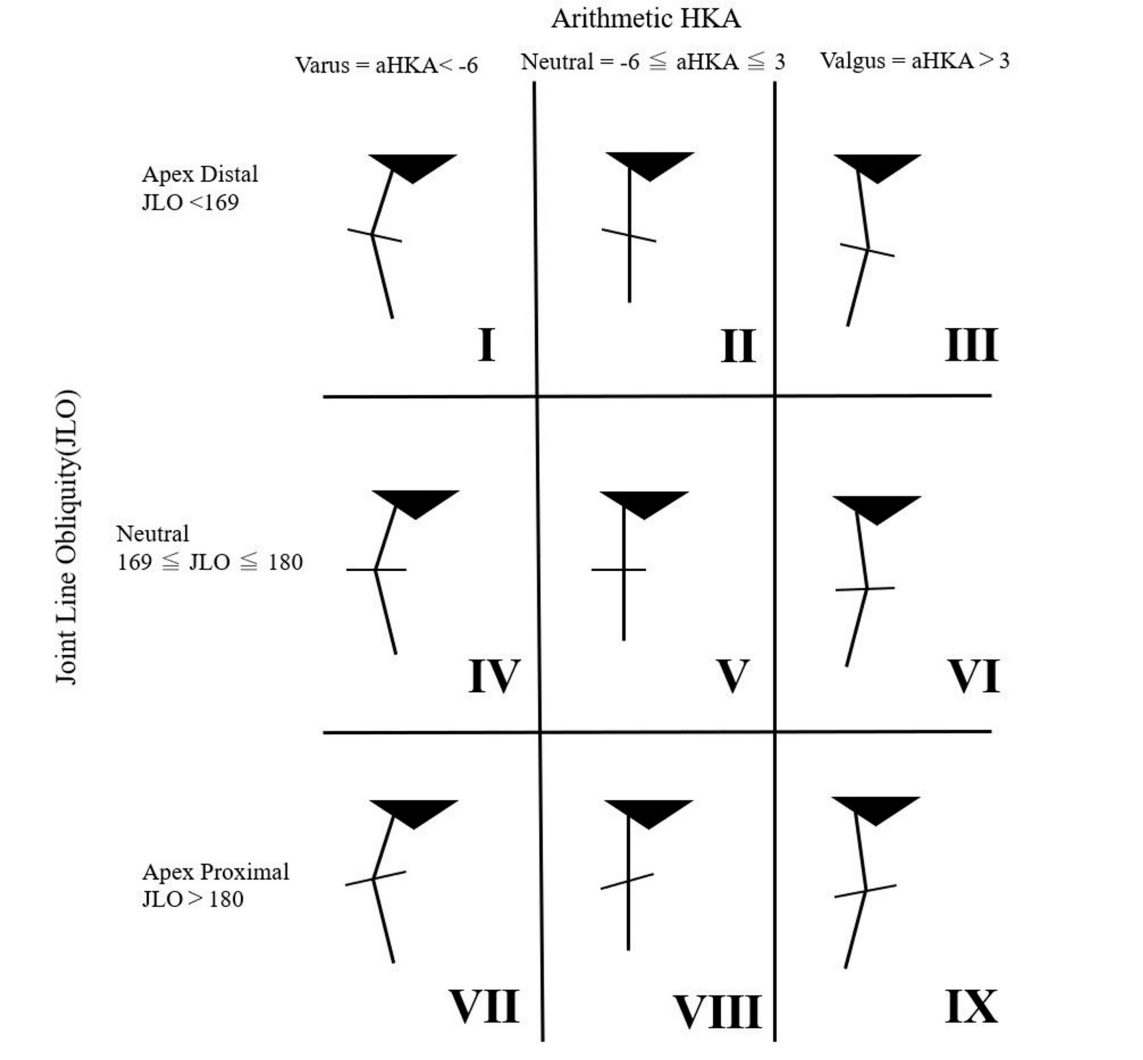
Restriction boundary-based coronal plane alignment of the knee The aHKA was set at 6° varus to 3° valgus according to the report of Winnock et al. [16], and regarding the JLO, a range of 169° to 180° was set according to MPTA and LDFA. This figure was adapted from Vendittoli et al. [12]. aHKA, arithmetic hip-knee-ankle angle; HKA, hip-knee-ankle angle; JLO, joint line obliquity; LDFA, lateral distal femoral angle; MPTA, medial proximal tibial angle

As reported previously [9,16], we modified the original CPAK by adjusting MPTA up to 6° varus, but avoided a valgus inclination (MPTA: 6° varus to 0°) (Figure 4). Regarding LDFA, which was not mentioned in the report by Winnock de Grave et al., we set the boundary as 5° valgus and, again, we set the boundary as 5° valgus to 0° to avoid a varus LDFA because it might not be natural and implies a femoral side bone loss. We considered avoiding a varus LDFA, but we did not manipulate the femoral side at all because we did not use any computer-aided devices that enable a controllable femoral cut referring the mechanical axis. We modified the original CPAK by adjusting LDFA from 5° valgus to 0°. The aHKA was set at 6° varus to 3° valgus according to the report of Winnock et al. [16]. Regarding the JLO, a range of 169° to 180° was set according to MPTA and LDFA.

Statistics analysis

LDFA, MPTA, aHKA, and JLO were compared pre- and postoperatively using the paired t-test. These distributions were also compared using the chi-square test. Distribution of original CPAK and Rb-CPAK were compared using Fischer's exact test. All statistical analyses were performed using easy R (EZR, Jichi Medical University, Shimotsuke, Japan) [17] running on R (R Foundation for Statistical Computing, Vienna, Austria). Post hoc power analysis was conducted using G*Power.

## Results

Distribution of original and Rb-CPAK

The distribution of pre- and postoperative original CPAK and Rb-CPAK is shown in Table 1. By modifying CPAK, both pre- and postoperative distribution was significantly different between original CPAK and Rb-CPAK (preoperative original CPAK vs Rb-CPAK p < 0.0001, postoperative original CPAK vs Rb-CPAK p < 0.0001). Regarding preoperative phenotypes, in original CPAK, 94 (66%) cases were classified as type I preoperatively and 21 (15%) cases were classified as type II. The most common type was V (59 cases [41%]) followed by IV (46 cases [32%]) preoperatively, and 98 (69%) cases were classified as V postoperatively in Rb-CPAK (Table 1). Only 35 (37%) cases of type I in the original CPAK were the same type postoperatively. Meanwhile, the 45 (76%) cases that were type V did not change type postoperatively.

**Table 1:** The distribution of pre- and postoperative original CKAP and Rb-CPAK CPAK, coronal plane alignment of the knee; Rb-CPAK, restriction boundary-based coronal plane alignment of the knee

	Original CPAK			Rb-CPAK		
	Preoperative	Postoperative	Same	Preoperative	Postoperative	Same
Ⅰ	94 (66%)	38 (27%)	35 (37%)	12 (8%)	0 (0%)	0 (0%)
Ⅱ	21(15%)	39 (27%)	9 (43%)	12 (8%)	5 (3%)	3 (25%)
Ⅲ	14 (10%)	17 (12%)	5 (36%)	1 (0%)	0(0%)	0 (0%)
Ⅳ	9 (6%)	22 (15%)	5 (56%)	46 (32%)	9 (6%)	8 (17%)
Ⅴ	3 (2%)	20 (14%)	0 (0%)	59 (41%)	98 (69%)	45 (76%)
Ⅵ	1 (0%)	5 (3%)	1 (100%)	10 (7%)	12 (8%)	1 (10%)
Ⅶ	1 (0%)	1 (0%)	0 (0%)	0 (0%)	5 (3%)	0 (0%)
Ⅷ	0 (0%)	0 (0%)	0 (0%)	3 (2%)	13 (9%)	1 (0%)
Ⅸ	0 (0%)	1 (0%)	0 (0%)	0 (0%)	1 (0%)	0 (0%)
Total	143 (100%)			143 (100%)		

Leg alignment (aHKA)

Regarding leg alignment, by widening the width of the HKA (from 6° varus to 3° valgus), preoperative original CPAK classification showed that 72% of the patients had a varus knee (aHKA less than -2), while preoperative Rb-CPAK modified it to 40% varus knee (aHKA less than -6). Furthermore, most of the lower limb alignment was neutral in postoperative Rb-CPAK classification (116 knees of 143 knees). The need for restriction of MPTA and LDFA by category of aHKA in original CPAK and Rb-CPAK is shown in Tables 2, 3. When determining the need for MPTA and LDFA restrictions separately, MPTA required more modifications than LDFA. Looking at the parts of LDFA and MPTA that did not require restriction, all Rb-CPAKs belonged to the part where aHKA was neutral (Table 3).

**Table 2:** Number of MPTA and LDFA restrictions required by aHKA in original CPAK aHKA, arithmetic hip-knee-ankle angle; CPAK, coronal plane alignment of the knee; LDFA, lateral distal femoral angle; MPTA, medial proximal tibial angle

	Preoperative aHKA (original CPAK)			Total
	aHKA < -2	-2 ≦ aHKA ≦ 2	aHKA > 2	
Knees	114	24	15	143
MPTA requires correction (MPTA < 84, 90 < MPTA LDFA is not restriction)	39	0	2	41
LDFA requires correction (LDFA < 85, 90 < LDFA MPTA is not restriction)	14	2	9	25
Both require correction	33	5	2	40
LDFA and MPTA are within range	18	17	2	37

**Table 3:** Number of MPTA and LDFA restrictions required by aHKA in Rb-CPAK aHKA, arithmetic hip-knee-ankle angle; CPAK, coronal plane alignment of the knee; Rb-CPAK, restriction boundary-based coronal plane alignment of the knee; LDFA, lateral distal femoral angle; MPTA, medial proximal tibial angle

	Preoperative aHKA (Rb-CPAK)			Total
	aHKA < -6	-6 ≦ aHKA ≦ 3	aHKA > 3	
Knees	58	74	11	143
MPTA require correction (MPTA < 84, 90 < MPTA LDFA is not restriction)	22	18	1	41
LDFA require correction (LDFA < 85, 90 < LDFA MPTA is not restriction)	6	11	8	25
Both require correction	30	8	2	40
LDFA and MPTA are within range	0	37	0	37

Joint line obliquity

We evaluated JLO without regard to aHKA. In original CPAK, 34 out of 37 knees were classified as apex distal even though the LDFA and MPTA were within range (Table 4). On the other hand, in Rb-CPAK, all 37 knees with neutral JLO had both LDFA and MPTA within range. However, among the cases with neutral JLO, we found 78 knees in which MPTA or LDFA required correction (Table 5).

**Table 4:** Evaluation of JLO without regard to aHKA in original CPAK aHKA, arithmetic hip-knee-ankle angle; JLO, joint line obliquity; LDFA, lateral distal femoral angle; MPTA, medial proximal tibial angle; Rb-CPAK, restriction boundary-based coronal plane alignment of the knee

	Preoperative JLO (original CPAK)			Total
	JLO < 177	177 ≦ JLO ≦ 183	JLO > 183	
Knees	129	13	1	143
MPTA require correction (MPTA < 84, 90 < MPTA LDFA is not restriction)	40	1	0	41
LDFA require correction (LDFA < 85, 90 < LDFA MPTA is not restriction)	18	7	0	25
Both require correction	36	3	1	40
LDFA and MPTA are within range	35	2	0	37

**Table 5:** Evaluation of JLO without regard to aHKA in Rb-CPAK aHKA, arithmetic hip-knee-ankle angle; JLO, joint line obliquity; LDFA, lateral distal femoral angle; MPTA, medial proximal tibial angle; Rb-CPAK, restriction boundary-based coronal plane alignment of the knee

	Preoperative JLO (Rb-CPAK)			Total
	JLO < 169	169 ≦ JLO ≦ 180	JLO > 180	
Knees	25	115	3	143
MPTA require correction (MPTA < 84, 90 < MPTA LDFA is not restriction)	15	26	0	41
LDFA require correction (LDFA < 85, 90 < LDFA MPTA is not restriction)	2	22	1	25
Both require correction	8	30	2	40
LDFA and MPTA are within range	0	37	0	37

LDFA and MPTA

**Figure 5:**
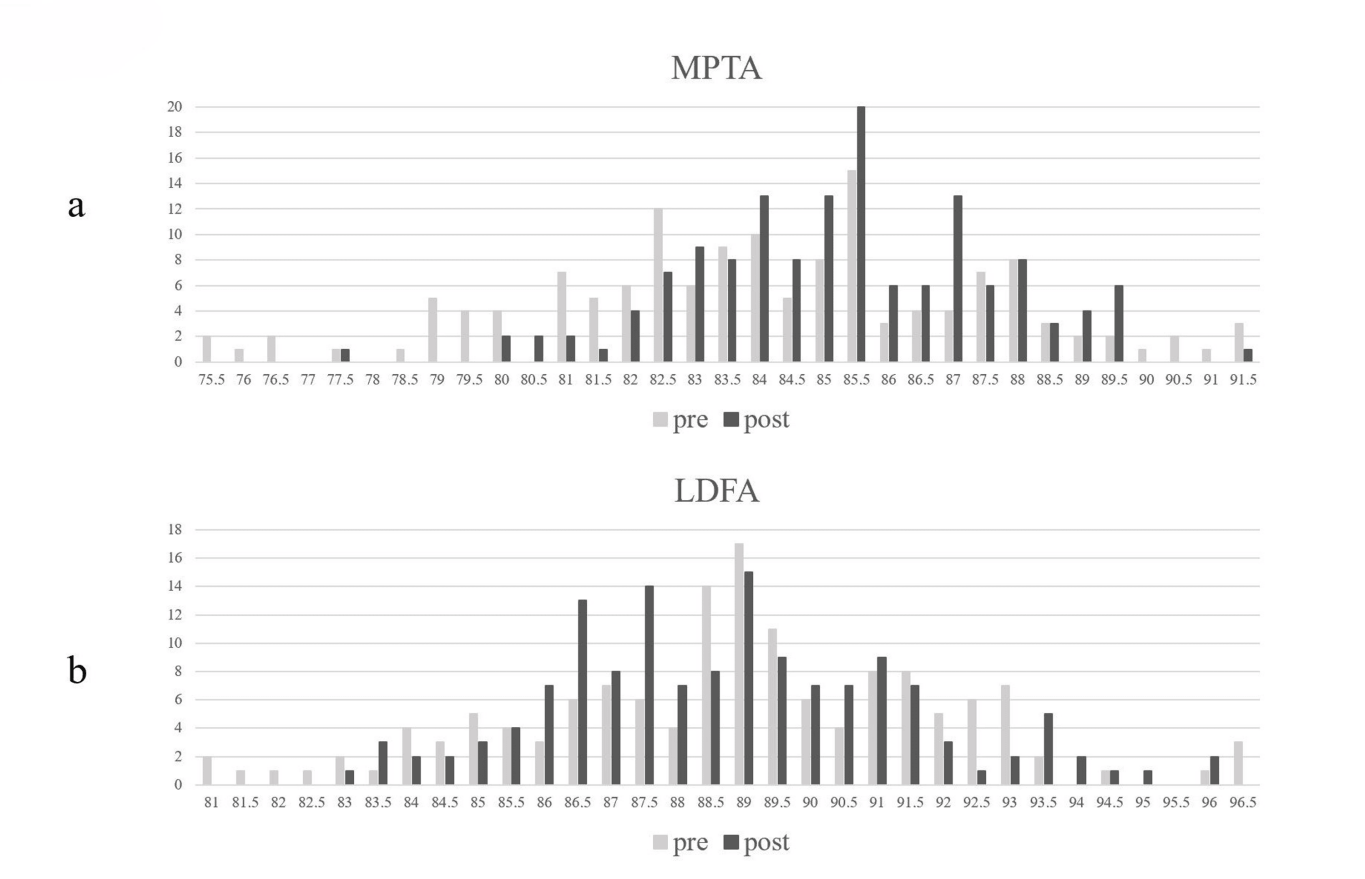
Pre- and postoperative the frequency distribution in MPTA and LDFA LDFA, lateral distal femoral angle; MPTA, medial proximal tibial angle

Mean pre- and postoperative MPTA were 83.9 ± 3.4 and 87.0 ± 2.3, respectively, showing a significant decrease of varus angulation (p < 0.0001). On the other hand, mean pre- and postoperative LDFA were 88.7 ± 3.1 and 88.5 ± 2.7, respectively. There was no significant difference between them (p = 0.4). The distribution of pre- and postoperative LDFA and MPTA is shown in Figure 5. The preoperative MPTA ranged widely between 75° and 92°, but the number of preoperative LDFA was the highest at 89° (Figure 5). The postoperative distribution of MPTA was different from preoperative distribution, whereas the pre- and postoperative distributions were similar in LDFA.

Effect of restriction

**Figure 6:**
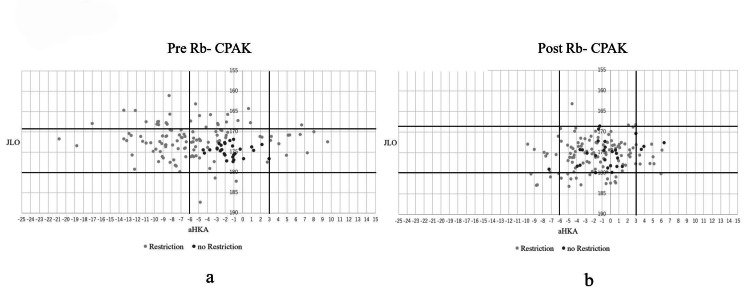
Cases with restrictions and the cases without restrictions plotting in Rb-CPAK The cases with restrictions and the cases without restrictions were plotted separately and applied to the categories of pre-Rb-CPAK. (a) Preoperative Rb-CPAK. (b) Postoperative Rb-CPAK. aHKA, arithmetic hip-knee-ankle angle; Rb-CPAK, restriction boundary-based coronal plane alignment of the knee

The cases with and without requirement of restriction were plotted separately in Figure 6. Preoperatively, all 28 knees without requirement of restriction were categorized in type V of Rb-CPAK. Postoperatively, 22 (71%) out of 28 knees fell into type V.

## Discussion

The most important findings of this study were that the distribution of original CPAK and Rb-CPAK are significantly different and that type V was most common in both pre- and postoperative Rb-CPAK. The original CPAK aims to express if a patient has a neutral leg alignment and JLO. Rb-CPAK, on the other hand, focuses on whether or not the patient has acceptable alignment for performing an rKA. It clearly shows whether a patient requires the restriction or not and whether rKA surgery has been performed properly or not. We believe that the Rb-CPAK is valuable not only for rKA but also for true KA, in which the component surface is same as the preoperative bone surface; therefore, the category is not changed in both CPAKs if the surgery is properly performed. The postoperative Rb-CPAK is expected to be categorized in type V after rKA-TKA and in the same category after true KA, regardless of the preoperative distribution of the knees.

Our results showed that more than 80% knees fell into the neutral range in Rb-CPAK, whereas only 41% of knees were categorized as neutral in original CPAK. This difference is due to difference in the setting of “neutral” between the classification systems. In the original CPAK, neutral is considered to be a nearly straight leg, whereas in Rb-CPAK, neutral is defined to be a safe zone in which no restriction is required. Although the safe zone can differ between restriction protocols, it can effectively preoperatively express if the restriction is required or not and can postoperatively express if the restoration was properly executed.

Similarly, JLO tended to be neutral postoperatively in Rb-CPAK compared with the original CPAK. This may be due to the setting of neutral range such as aHKA, but at least one of LDFA and MPTA fell out of the safe range; therefore, restriction is required in most knees. JLO depends on the relationship between LDFA and MPTA. If the LDFA are MPTA in same direction, e.g., LDFA: varus and MPTA: varus or LDFA: valgus and MPTA: valgus, the JLO is neutral (if MPTA is 85° and LDFA is 95°, then JLO is neutral at 180°). JLO is therefore insufficient for estimating the joint line for rKA-TKA, and consideration of individual LDFA and MPTA is necessary to control the joint line.

In this study, all knees that did not require restriction were classified as type V in the preoperative Rb-CPAK classification. Although 22 (79%) out of 28 knees were classified as type V, six (11%) knees fell out of the category. This might be due to the use of manual instruments. Moreover, we applied the restriction only for the tibial cut. The use of computer aided devices, such as robotics and patient-specific instrumentation, might increase the knees classified as type V. In restriction of the femoral side, controlling the thickness of the distal end of femur might be needed for feasible calipered restriction technique using conventional instruments. The Rb-CPAK can ascertain not only the necessity of restriction preoperatively but also the result of restriction, indicating the appropriateness of the procedure.

In this study, we used our restriction boundary that aHKA was set from 6° varus to 3° valgus, and JLO was set a range of 169° to 180°. Although some other boundaries have been advocated [18-20] (Table 6), why the boundaries are decided and if the boundaries are proper has not been reported. Most boundaries might be decided based on the outlier in terms of MA-TKA, which can cause implant failure. However, the outlier in MA-TKA and KA-TKA might be different because, unlike MA-TKA, proper gap balancing can be achieved even when the alignment is not neutral and mechanical in KA-TKA. MacDessi et al. [13] decided boundaries based on “normal” population. However, there is a lack of evidence that implant failure can increase if the leg and component alignment fall out of the normal distribution range. Further study is needed to find an evidence-based boundary, where if the alignment is out of the boundary, the implant failure will increase. The boundary might differ between races, ages, and genders.

**Table 6:** Comparison of other boundaries in Rb-CPAK aHKA, arithmetic hip-knee-ankle angle; JLO, joint line obliquity; LDFA, lateral distal femoral angle; MPTA, medial proximal tibial angle; Rb-CPAK, restriction boundary-based coronal plane alignment of the knee

	aHKA	LDFA	MPTA	Assumed range of JLO	Type Ⅴ pre/post (%)
Almaawi et al. [18]	-3 to 3	85 to 95	85 to 95	170-190	41/73
MacDessi et al. [19]	-5 to 4	86 to 93	86 to 93	172-186	34/70
McEwen et al. [20]	-6 to 3	84 to 83	84 to 93	168-176	34/48
Our hospital	-6 to 3	85 to 90	84 to 90	169-180	41/61

Limitations

Our study had some limitations. We performed measurements using a standing radiograph of the lower limb, which can differ depending on the rotation of the femur and tibia when standing in each case, and thus there may be measurement errors. To correct this situation, 3D analysis using computed tomography is desirable, but it is costly and can be difficult to perform with the patient standing. We excluded incorrect X-rays and cases of flexion contracture ≥15° but considered the effect of these limitations to be small. We set the restriction boundary for the femur but did not perform any correction osteotomy. Although correction is required for knees out of boundary, Rb-CPAK well outlined the shortage of our technique. Although restriction procedures have been reported regarding the tibial side, a reliable restriction technique for the femoral side should be established. In addition, the results of this study did not consider the clinical outcomes. Further research will evaluate the long-term results.

## Conclusions

By modifying CPAK, both pre- and postoperative distributions were significantly different between original CPAK and Rb-CPAK. The Rb-CPAK modification more effectively outlined knees that required restriction, and restriction was properly performed compared with the original CPAK. However, JLO does not effectively indicate if a knee require restriction or not; therefore, individual evaluation of LDFA and MPTA might be necessary.
